# Vegetable Oil Derived Solvent, and Catalyst Free “Click Chemistry” Thermoplastic Polytriazoles

**DOI:** 10.1155/2014/792901

**Published:** 2014-06-17

**Authors:** Michael C. Floros, Alcides Lopes Leão, Suresh S. Narine

**Affiliations:** ^1^Trent Centre for Biomaterials Research, Departments of Physics and Astronomy and Chemistry, Trent University, 1600 West Bank Drive, Peterborough, ON, Canada K9J 7B8; ^2^College of Agricultural Sciences, Sao Paulo State University, (UNESP), 18610-307 Botucatu, SP, Brazil

## Abstract

Azide-alkyne Huisgen “click” chemistry provides new synthetic routes for making thermoplastic polytriazole polymers—without solvent or catalyst. This method was used to polymerize three diester dialkyne monomers with a lipid derived 18 carbon diazide to produce a series of polymers (labelled C18C18, C18C9, and C18C4 based on monomer chain lengths) free of residual solvent and catalyst. Three diester dialkyne monomers were synthesized with ester chain lengths of 4, 9, and 18 carbons from renewable sources. Significant differences in thermal and mechanical properties were observed between C18C9 and the two other polymers. C18C9 presented a lower melting temperature, higher elongation at break, and reduced Young's modulus compared to C18C4 and C18C18. This was due to the “odd-even” effect induced by the number of carbon atoms in the monomers which resulted in orientation of the ester linkages of C18C9 in the same direction, thereby reducing hydrogen bonding. The thermoplastic polytriazoles presented are novel polymers derived from vegetable oil with favourable mechanical and thermal properties suitable for a large range of applications where no residual solvent or catalyst can be tolerated. Their added potential biocompatibility and biodegradability make them ideal for applications in the medical and pharmaceutical industries.

## 1. Introduction

Dwindling reserves and price increases are driving research for alternatives to petrochemical feedstocks used in materials and fuels. Polymers derived from nonpetrochemical sources are becoming more viable and economical as improvements in feedstock, processing, and technology are developed. One area receiving significant attention is that of polymers derived from vegetable oils. Vegetable oils are abundant, renewable, and possess favourable properties including biofragmentability and biocompatibility compared to petrochemical analogs [1]. They can be relatively easily converted and modified into an ever-increasing variety of materials with diverse functional properties. Triglycerides, fatty acids, alcohols, and other modified vegetable oil derived monomers have been used to create a large range of thermoset and thermoplastic polymers, such as polyesters [2], polyurethanes [3, 4], epoxides, and polyethers as well as thiol-ene and triazole based polymers [5–7]. Research targeted at improving the properties and scope of vegetable oil derived polymers is rapidly growing and involves multidisciplinary approaches.

Environmentally favourable chemistries using benign conditions and producing little or no byproducts are the subject of extensive research. Polymers are generally synthesized using solvents and metal catalysts. Although tolerated at small levels for commodity polymers, some solvents, such as DMF and 1,2-dichlorobenzene, and metal catalysts, are toxic and potentially carcinogenic and are very hard to remove. This is particularly critical in biomedical and pharmaceutical applications where solvent and catalyst free synthesis routes are highly preferred [8]. More generally, it is important to limit the use of catalyst and solvent because of toxicity and environmental concerns.

Recently, azide-alkyne Huisgen 1,3-dipolar cycloaddition has begun to find its way into the repertoire of tools available to green chemists [6]. This reaction employs reactive, “spring loaded”, terminal azide, and alkyne groups to afford 1-4-, or 1-5-substituted, 1,2,3-triazoles—depending on the catalyst. Uncatalyzed reactions produce a mixture of the 1,4- and 1,5-substituents, with the 1,4-[1,2,3-triazole] regioisomer favoured by a ratio around 2 : 1 [9]. The interest in the azide-alkyne Huisgen cycloaddition reaction was renewed in the early 2000s after Barry Sharpless popularized the copper catalyzed variant and classified it as one type of “click” chemistry, a reaction which can rapidly and easily produce a wide variety of functional materials having high yields, easy purifications, high atomic economy, and mimics nature [10, 11]. Some advantages of azide-alkyne “click” reactions include the lack of requirement for solvent or catalyst, complete consumption of reactants with no byproduct or gas formed, and unique properties of the triazole moiety [10, 12, 13]. Triazole containing compounds have been found to possess antimicrobial and self-extinguishing characteristics—these are desirable characteristics in polymers [12]. Polymerizations using azide-alkyne “click” chemistry have been applied to various types of monomers, including vegetable oil triglycerides, forming a series of cross-linked and thermoset polymers [6, 14]. The utility of azide and alkyne “spring-loaded” reactants as building blocks for complex molecules like polymers is beginning to be realized, opening a door to producing macromolecules with novel and unique functionality [15].

The goal of this work was to create lipid derived thermoplastic polymers using the commonly available and economical feedstocks: succinic acid, azelaic acid, and octadec-9-enedioic acid—all of which are produced from renewable resources [16]. Succinic acid is produced by the bacterial fermentation of carbohydrates in a green process which consumes CO_2_. It is a common commercial feedstock and can be used to produce industrially important products, such as 1,4-butanediol and polyesters [17]. Worldwide production was approximately 300 kt in 2010 and global demand has been estimated at 30 mt/year, while optimizations in production may lower costs to $0.36/kg [18, 19]. Both azelaic acid and octadec-9-enedioic acid are produced from oleic acid, closely tying their price to that of the feedstock. Azelaic acid is produced on a commercial scale by the ozonolysis of oleic acid and used as a plasticizer and in polymers, including nylon 6,9 [20]. Octadec-9-enedioic acid is produced by the self-metathesis of oleic acid or by biotransformation of 18 carbon fatty acids using modified yeast fermentation [21, 22]. These renewable monomeric feedstocks provide comparable properties to conventional petroleum derived products and are of growing importance as their supply improves and the price of petroleum continues to increase.

The intent of varying the chain length of the monomer was to establish the degree of dependence on monomer chain length and degree of asymmetry. Furthermore, the relationship between even and odd chain length monomers, known as the odd-even effect, can be probed by comparing the properties of the azelaic acid based monomer (9 carbons) with the two other monomers, each having an even number of carbon atoms. Polyesters with even carbon numbered monomers demonstrate higher melting temperatures and enthalpies of fusion than their odd carbon number analogues [23, 24].

In the present work, three thermoplastic polytriazoles from diester dialkynes and a diazide monomer derived from oleic acid (with the exception of the C4 dialkyne, which is produced from succinic acid, a product of bacterial fermentation of carbohydrates) were synthesized and characterized. The numbers of carbon atoms in the diester dialkyne monomers are 4, 9, and 18, with 18 for the diazide monomer. This work presents a new route to the creation of melt-processable polymers from renewable vegetable oils without residual solvent or catalyst. Furthermore, because of the presence of unsaturated sites in the monomers, it may be possible to perform polymerization on a suitable substrate* in situ* and further modify the unsaturation to afford a potentially biocompatible polymer with easily tunable functionalities.

## 2. Experimental

### 2.1. Materials

Azelaic acid (98%), oleic acid (85%), sodium azide (99%), propargyl alcohol (99%), 4-toluenesulfonyl chloride (98%), triethyl amine (99%), succinic acid (99%), Grubbs catalyst 2nd generation, LiAlH_4_ (95%), N,N′-dicyclohexylcarbodiimide (DCC) (99%), and 4-dimethylaminopyridine (DMAP) (99%) were purchased from Sigma-Aldrich and used as received. (*E*)-octadec-9-ene-1,18-diol and (*E*)-1,18-octadec-9-endiacid were synthesized using a previously reported procedure [3], that is, the olefin metathesis of oleic acid followed by the subsequent reduction of the diacid into the dialcohol.

## 3. Methods

### 3.1. Synthesis of Monomers

**Scheme 1:**
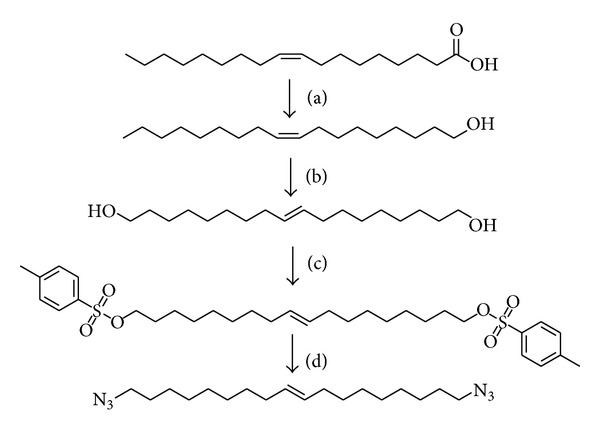
Synthesis of vegetable derived diazide monomer. (a) Grubbs 2nd gen, neat, 45°C, 12 h. (b) LiAlH_4_, THF, 0°C → RT, 4 h. (c) TsCl, TEA, DMAP, CH_2_Cl_2_ 0°C → RT, 6 h. (d) NaN_3_, DMSO, H_2_O, 100°C, 12 h.

**Scheme 2:**
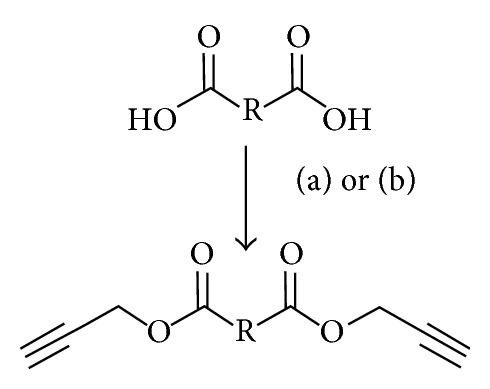
Synthesis of vegetable derived dialkyne monomers. (a) For saturated dicarboxylic acids: propargyl alcohol + 5 drops H_2_SO_4_, toluene, 100°C, 12 h. (b) For unsaturated dicarboxylic acid: DCC/DMAP, CHCl_3_, 50°C, 12 h.

Oleic acid was converted into a dicarboxylic acid through olefin metathesis, reduced into a dialcohol, tosylated, and finally azidated into a diazide (Scheme 1). Three diester dialkynes monomers were subsequently synthesized from dicarboxylic acids of different carbon lengths—(*E*)-1,18-octadec-9-endiacid (C18), azelaic acid (C9), and succinic acid (C4) through either Steglich or Fischer esterification (Scheme 2).

*(E)-Octadec-9-ene-1,18-diyl bis(4-methylbenzenesulfonate)* **(1)**. (*E*)-Octadec-9-ene-1,18-diol (12.0 g, 42.2 mmol) was added to a round bottom flask kept in an ice bath (0°C). 4-Toluenesulfonyl chloride (18.2 g, 95.4 mmol) dissolved in 150 mL of CH_2_Cl_2_ and was added dropwise, then 0.30 g (2.5 mmol) of DMAP and 12.0 g (119 mmol) of triethylamine dissolved in 20 mL CH_2_Cl_2_ was added dropwise. The reaction was allowed to warm to room temperature and proceeded until complete consumption of the starting material, as determined by TLC (approximately 6 hours, Rf = 0.30 5 : 1 hexane/ethyl acetate). The contents of the flask were diluted with 150 mL CH_2_Cl_2_ and washed with water (3 × 150 mL), 2.5 N HCl solution (3 × 150 mL), 4% aqueous sodium bicarbonate (3 × 150 mL), and finally saturated NaCl brine (3 × 150 mL). The organic layer was dried with anhydrous Na_2_SO_4_ and concentrated under reduced pressure yielding 22.3 g of slightly yellow crystal. The solid product was purified by recrystallization from hexanes twice and dried in a vacuum oven. 18.0 g of a white solid was recovered (72% yield). ^1^H-NMR (500 MHz in CDCl_3_): *δ* 1.21 (m, 20H), 1.62 (p, 4H), 1.94 (m, 4H), 2.45 (s, 6H), 4.01 (t, 4H), 5.36 (dtt, 2H), 7.34 (d, 4H), and 7.79 (d, 4H).

*(E)-1,18-Diazidooctadec-9-ene* **(2)**. 8.2 g of (**1**) was added to a round bottom flash equipped with a reflux condenser. 90 mL of DMSO was added and 12.0 g of NaN_3_ dissolved in 10 mL deionized water was introduced with stirring. Immediately following addition, the reaction turned slightly yellow and was warm to the touch. It was heated to 100°C and stirred for 12 hours at this temperature. Upon completion, the mixture was cooled to room temperature and poured into 250 mL of ice water to precipitate the product and separate the DMSO into the aqueous phase. The organic layer was extracted with ethyl acetate (3 × 150 mL) and washed with water (2 × 150 mL) and NaCl brine (2 × 150 mL). The organic phase was dried over Na_2_SO_4_ and concentrated under reduced pressure. It was then purified by column chromatography on silica gel using a 50 : 1 ratio of hexanes to ethyl acetate to afford a colourless liquid (4.3 g, yield = 93%). ^1^H-NMR (500 MHz in CDCl_3_): *δ* 1.29 (m, 20H), 1.59 (p, 4H), 1.96 (m, 4H), 3.25 (t, 4H), and 5.38 (dtt, 2H).

*(E)-Di(prop-2-yn-1-yl) octadec-9-enedioate* **(3)**. (*E*)-1,18-octadec-9-ene diacid (20.0 g, 64 mmol) was added to a round bottom flask with 200 mL of chloroform and heated to 50°C. The mixture was stirred until it became clear and then 7.89 g propargyl alcohol (140 mmol) was added to the flask. N,N′-dicyclohexylcarbodiimide (28.9 g, 140 mmol) in 50 mL CHCl_3_ was added dropwise with 0.5 g 4-dimethylaminopyridine (DMAP) over 30 min. A reflux condenser was then equipped and the solution was stirred for 12 hours at 50°C then cooled to room temperature and filtered over celite to remove precipitated dicyclohexylurea. The chloroform was removed under reduced pressure and the compound was dissolved in hot hexanes and filtered while hot over celite. The product was recrystallized from hexanes twice, with only the* trans* product recovered and dried in a vacuum oven overnight to yield 20.4 g of a white powder (yield: 82%). ^1^H-NMR (500 MHz in CDCl_3_): *δ* 1.31 (m, 16H), 1.65 (quin, 4H), 1.97 (m, 4H), 2.36 (t, 4H), 2.47 (t, 2H), 4.68 (d, 4H), and 5.38 (dtt, 2H).

*Di(prop-2-yn-1-yl) nonanedioate* **(4)**. Azelaic acid (10.0 g, 53 mmol) was added to a round bottom flask with 75 mL toluene and heated to 100°C. The mixture was stirred until the solution became clear then 20 mL (347 mmol) of propargyl alcohol was added. Five drops of concentrated sulfuric acid in 2 mL toluene was slowly added to the mixture and the solution was stirred for 12 hours. The reaction was cooled to room temperature and then it was diluted with an additional 75 mL toluene and washed with water (3 × 100 mL), then a sodium bicarbonate solution, (3 × 100 mL), and finally saturated NaCl brine (2 × 100 mL). The organic layer was dried with anhydrous sodium sulfate and concentrated under reduced pressure. The product was purified by vacuum distillation at 150 mtorr and the fraction boiling between 133°C and 136°C collected as a colourless liquid (11.60 g, yield: 83%). ^1^H-NMR (500 MHz in CDCl_3_):*δ* 1.32 (m, 6H), 1.63 (m, 4H), 2.35 (t, 4H), 2.47 (t, 2H), and 4.67 (d, 4H).

*Di(prop-2-yn-1-yl) succinate* **(5)**. Succinic acid (10.0 g, 84.7 mmol) was added to a round bottom flask with 75 mL toluene and heated to 100°C. The mixture was stirred until the solution became clear and 20 mL propargyl alcohol (357 mmol) was added. 5 drops of concentrated sulfuric acid in 2 mL toluene was then slowly added to the mixture, and the solution was stirred for 12 hours. The reaction was cooled to room temperature, then the mixture was diluted with an additional 75 mL toluene and washed with water (3 × 100 mL), then a sodium bicarbonate solution (3 × 100 mL) and finally with saturated NaCl brine (2 × 100 mL). The organic layer was dried with anhydrous sodium sulfate and concentrated under reduced pressure. The product was purified by vacuum distillation at 160 mtorr and the fraction boiling between 89°C and 91°C was collected as a colourless liquid (14.06 g, yield 85%). ^1^H-NMR (500 MHz in CDCl_3_): *δ* 2.48 (t, 2H), 2.69 (s, 4H), 4.69 (d, 4H).

### 3.2. Polymerization

Equal molar ratios of a dialkyne monomer and a diazide monomer were added to a polytetrafluoroethylene (PTFE) round bottom flask. The flask was purged with nitrogen for 15 minutes then placed in an oil bath under nitrogen protection and heated from room temperature to 110°C gradually over a 2-hour period to prevent thermal decomposition of the reactants in the exothermic polymerization. It was then stirred for an additional 20 hours at this temperature. The polymer product was cooled to room temperature and cast into films by melting at 170°C inside a stainless steel mold (70 × 30 × 0.6 mm) covered with a sheet of PTFE. The molten sample was pressed on a Carver 12-ton hydraulic heated bench press (Model 3851-0, Wabash, IN, USA). Three metric tons of pressure were applied isothermally for 15 min to the mold. The sample was then cooled to 20°C over 30 min by pumping coolant through the plates on the hydraulic press period using a Julabo temperature controlled circulating bath (Model FP50-ME, Seelbach, Germany). The polymer film was stored in a sealed container until analysis.

### 3.3. Characterization Techniques

The ^1^H spectra were recorded on a Varian Unity-INOVA at 499.695 MHz. ^1^H chemical shifts were internally referenced to CDCl_3_ (7.26 ppm) for spectra recorded in CDCl_3_ and 7.19 ppm for spectra recorded in 1,2-dichlorobenzene-*d*_4_. All spectra were obtained using an 8.6 *μ*s pulse with 4 transients collected in 16,202 points. Datasets were zero-filled to 64,000 points and a line broadening of 0.4 Hz was applied prior to Fourier transforming the sets. The spectra were processed in ACD Labs NMR Processor, version 12.01.

In a typical ^1^H NMR of the monomers, vinylic protons −C**H**=**H**C= present around *δ* 5.38 ppm and allylic −C=CH−C**H**_**2**_− at *δ* 1.97. In the alkyne monomers, the propargylic proton −C(=O)OCH_2_C*≡*C**H** appears at *δ* 2.47-2.48 ppm, and the −C(=O)OC**H**_**2**_C*≡*CH appears at *δ* 4.67–4.69 ppm. The ester −C**H**_**2**_C(=O)O− occurs at *δ* 2.35-2.36 for the C9 and C18 alkynes but is shifted to *δ* 2.69 ppm in the C4 monomer due to the close proximity of the ester groups to each other. In the tosylate intermediate, shifts at *δ* 7.34 ppm and *δ* 7.79 ppm both correspond to different sets of protons on the aromatic ring and the benzylic −Bz−C**H**_**3**_ presents at *δ* 2.45. The tosylate ester influences both the −C**H**_**2**_OS− and −C**H**_**2**_CH_2_OS−, with shifts appearing at *δ* 4.01 and 1.62 ppm, respectively. Furthermore, in the azide monomer, we see a disappearance of the indicative shifts of the tosyl group. The −C**H**_**2**_N_3_ presents at *δ* 3.25 ppm and –C**H**_**2**_CH_2_N_3_ at 1.59 ppm. The remaining alkane −CH_2_C**H**_**2**_CH_2_- protons fall between *δ* 1.21 and 1.45 ppm.

Thermogravimetric Analysis was conducted on a TGA Q500 (TA Instruments, Newcastle, DE, USA). The sample (10–20 mg) was heated from room temperature to 600°C at a rate of 10.0°C/min under a nitrogen flow of 60.0 mL/min. Calorimetry studies were performed following ASTM E1356 standard on a DSC Q200 (TA Instruments, Newcastle, DE, USA.) equipped with a refrigerated cooling system. The sample (5-6 mg) was initially heated to 180°C for 10 min to remove thermal history and then cooled at a rate of 5.0°C/min down to −80°C, where it was held isothermally for 5 min. Finally the sample was heated from −80°C to 180°C at a rate of 5.0°C/min.

Dynamic mechanical analysis (DMA) measurements were carried out following ASTM D7028 standard on a Q800 (TA Instruments, Newcastle, DE, USA.) analyzer equipped with a liquid nitrogen cooling system. The sample was cut into a rectangular shape (17.5 mm × 12.0 mm × 0.60 mm) and was analyzed in the single cantilever bending mode. It was heated at a constant rate of 1.0°C/min from −100°C to 40°C. The measurements were performed at a frequency of 1 Hz and fixed oscillation displacement of 15 *μ*m.

Tensile tests were performed according to ASTM D882 standard using a mechanical analyser (Texture Technologies Corp, NJ, USA.) equipped with a 25-kg load cell. The film was die-cut by an ASTM D638 type V cutter. The sample was stretched at a rate of 60 mm/min from a gauge of 35 mm at room temperature.

ATR-FTIR spectra were collected using a Thermo Scientific Nicolet 380 FTIR spectrometer (Thermo Electron Scientific Instruments LLC, USA) fitted with a PIKE MIRacle attenuated total reflectance (ATR) system (PIKE Technologies, Madison, WI, USA). 0.60 mm thick polymer samples were placed onto the ATR crystal and pressed by a mechanical arm. Spectra of the polymer films were acquired over a scanning range of 500–4000 cm^−1^ for 128 repeated scans at a spectral resolution of 0.4821 cm^−1^. 16 repeated scans were used for monitoring the polymerization. The data was background corrected and baselined by the machine software (EZ OMNIC, version 7.3) and exported in spreadsheet format for graphing.

The crystalline structure and relative crystallinity of the polymers were examined by wide-angle X-ray diffraction (WAXD) using an EMPYREAN diffractometer system (PANanalytical, Netherlands) equipped with a filtered Cu–K *α* radiation source (*λ* = 1.540598 Å) and a PIXcel^3D^ detector. The scanning range was 2.0062°–30.000° (2*θ*) with a step size of 0.0263°. Data analysis was performed using PANalytical's X'Pert HighScore 3.0.4 software. The relative degree of crystallinity was estimated according to a well-established procedure [27]. The percentage degree of crystallinity (*X*_*C*_) is calculated by

(1) $$\begin{array}{c} X_{C}=100\times \frac{A_{C}}{A_{C}+A_{A}}, \end{array}$$

where *A*_*C*_ is the area under the resolved crystal diffraction peak contribution and *A*_*A*_ is the area of the amorphous contribution.

## 4. Results and Discussion

Vegetable derived dicarboxylic acids with *n* = 4, 9, and 18 carbon atoms were transformed into a dialkyne terminated diester monomers. The length of the dialkyne was varied by choosing three economically priced and plant derived dicarboxylic acids: succinic acid (C4), azelaic acid (C9), and octadec-9-enedioic acid (C18). The choice of ester linkages was made because they are simpler to synthesize from carboxylic acids, which are readily manufactured from vegetable triglycerides on a commercial scale. Ester linkages have also been shown to add biodegradability to polymers [28]. The diazide monomer, (*E*)-1,18-diazidooctadec-9-ene, which was prepared from the same C18 diacid, has 18 carbon atoms separating two high energy (azide) nitrogen groups. The significance of this is that the ratio of 3 carbon to 1 nitrogen atoms fulfills an important safety consideration for azides. This ratio is generally accepted as sufficient to prevent the rapid decomposition or explosion of azide groups and to allow safe storage and processing [29].

### 4.1. Polymerization and Characterization of the Polymers

In solvent polymerization, the solvent can aid in dissipating heat of exothermic events, which was not possible in this system. To circumvent this issue, the monomers were gradually oligomerized by heating to the polymerization temperature (110°C) over 2 hours with stirring. The viscosity increased gradually as oligomerization proceeded allowing prolonged mixing (which did not occur with rapid heating). Characterization of the polymer molecular weights was not possible due to poor solubility in common solvents, including DMF, DMSO, CHCl_3_, CH_2_Cl_2_, THF, 1,2-dichlorobenzene, *N*-methyl-2-pyrrolidone, methanol, 2-propanol, diethyl ether, ethyl acetate, acetone, hexanes, and acetonitrile.

**Figure 1:**
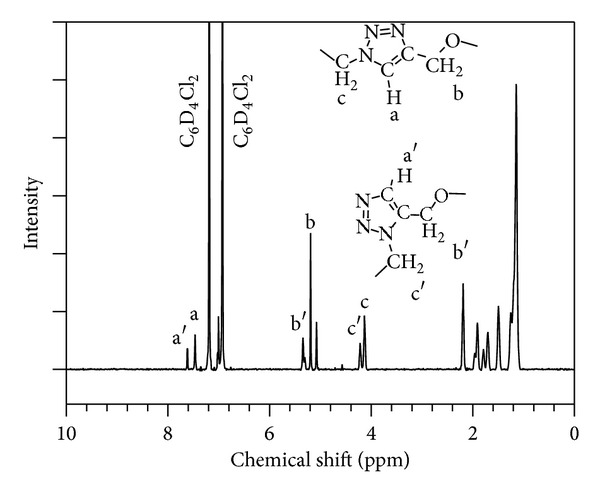
Select ^1^H NMR spectra of C18C9 showing labelled peaks for the 1,5 (a′, b′, and c′) and 1,4-substituted (a, b, and c) 1,2,3-triazoles.

^1^H NMR of the polymers in 1,2-dichlorobenzene-*d*_4_ was run at 45°C for improved polymer solubility. This temperature was a good compromise between increasing solubility and thermal noise. ^1^H NMR spectrum of C18C9, representative of the C18C*n* polymers, is shown in Figure 1 (A magnified proton NMR spectra of the polymer C18C9 from Figure 1 showing enhanced resolution of the triazole region shifts is provided in Figure S1 in the Supplementary Material and available online at http://dx.doi.org/10.1155/2014/792901). The limited solubility of the polymer caused significant line broadening of the NMR peaks, but the characteristic peaks for both the 1,4-(at *δ* 7.47) and the 1,5-substituted 1,2,3-triazoles (at *δ* 7.62) were observed in all the polymer samples. As can be seen in Figure 1, no signals were detected for characteristic protons of the azide (–C**H**_**2**_N_3_, *δ* 3.25) or alkyne (–C*≡*C–**H,** *δ* 2.46) groups on the monomers, indicating complete conversion. The ratio of the 1,4- to the 1,5- substitution estimated by integrating the area of each triazole C–H peak was 2 : 1.

**Figure 2:**
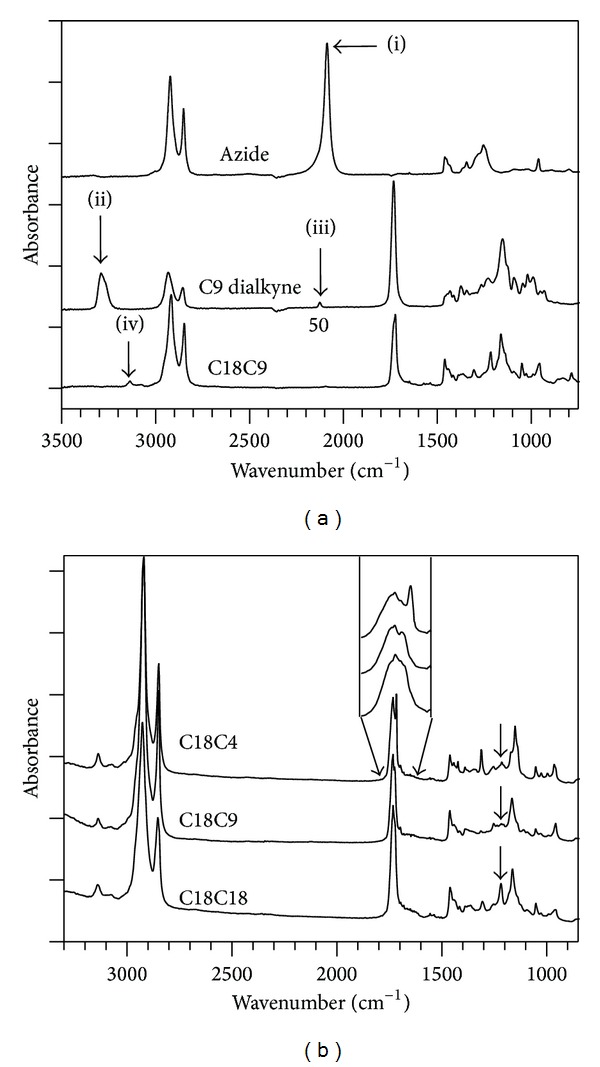
(a) ATR-FTIR of the azide monomer (characteristic peak i, 2091 cm^−1^), alkyne monomer (characteristic peaks ii, 3290 and iii, 2128 cm^−1^) and polymer C18C9 showing formation of a triazole (characteristic peak iv, 3144.5 cm^−1^). (b) FTIR spectra of the C18C4, C18C9, and C18C18 polymers. Arrow points to the triazole self-association absorption peak at 1222 cm^−1^. Insert is a higher resolution zoom into the carbonyl region.

Selected ATR-FTIR spectra obtained during the monitoring of the polymerization of C18C18 are shown in Figure 2(a). ATR-FTIR spectra of the polymers are shown in Figure 2(b). The characteristic azide peak (–**N=N=N**) (i, 2091 cm^−1^, in Figure 2(a)) and alkyne peaks (*≡* **C**–**H**, and –**C*≡*C**) (ii, 3290 and iii, 2128 cm^−1^ in Figure 2(a), resp.) which were present in the spectra of the starting materials were absent from the spectra of the polymers. In fact, as the reaction progressed, these peaks decreased in intensities and disappeared after 20 hours at 110°C. Concomitantly, the characteristic triazole ring peak (at 3142 cm^−1^) [30] appeared after 2 hours reaction time and increased steadily in intensity until the monomers were completely reacted.

The triazole group's C–H bond is known to be a hydrogen bond donor, resulting from the increased electronegativity of carbons within the triazole ring [31]. The sharp peak at 1736 cm^−1^ in all the polymers represents the free ester carbonyl C=O stretch. Only one peak representing the ester carbonyl C=O stretch is visible in the FTIR of C18C18 and a second peak was observed at 1730 cm^−1^ and 1720 cm^−1^ for C18C9 and C18C4 (see insert in Figure 2(b)), respectively. The second peak emerged as a shoulder for C18C9 and as a well-resolved peak for C18C4. The splitting or shifts in the ester carbonyl C=O stretching peak are a common indicator of hydrogen bonding in polymers [32]. The shift in wavenumber and increased magnitude of the split peak suggest that a larger amount of carbonyl groups are hydrogen bound in C18C4.

**Figure 3:**
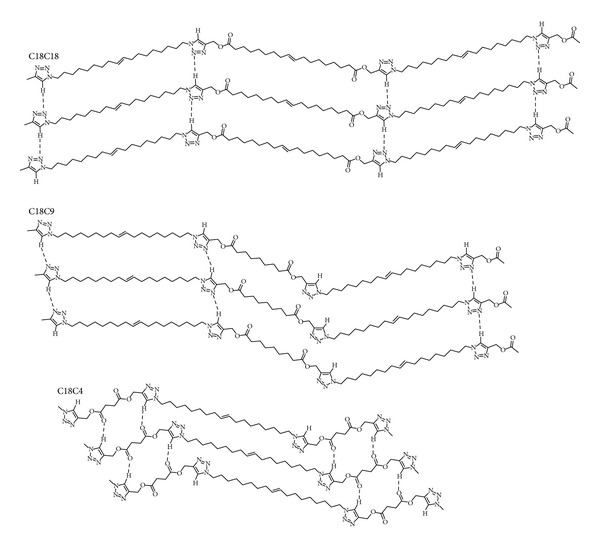
Schematic representation of the structure and hydrogen bonding of the polymers.

The peak at 1222 cm^−1^ was very intense in C18C18 compared to the other two polymers. It is characteristic of triazole self-association, where triazoles hydrogen bond to each other [33] suggesting that hydrogen bonding is occurring between the triazole rings of C18C18 (N–H*·* *·* *·*N bonding, Figure 3). Furthermore, the lack of a carbonyl peak splitting in this polymer suggests that hydrogen bonding is predominantly based on a triazole self-association mechanism. The 1222 cm^−1^ peak is absent for the C18C9 polymer and small in C18C4, indicating that a minority of the hydrogen bonding in these two polymers occurs through self-association.

The hydrogen bonding structures which are consistent with the FTIR data of the polymers are presented in Figure 3. In C18C18, hydrogen bonding is occurring primarily between triazole groups whereas in C18C9 and C18C4, it occurs predominantly between the triazole and carbonyl groups (Figure 3). Note that in C18C9, some of the hydrogen bond donating groups (triazole C–H) are unable to participate in hydrogen bonding due to their relative orientation, a result of the odd carbon number of its 9 carbon diester repeating unit, giving rise to the known odd-even effect.

### 4.2. Thermal Properties

**Figure 4:**
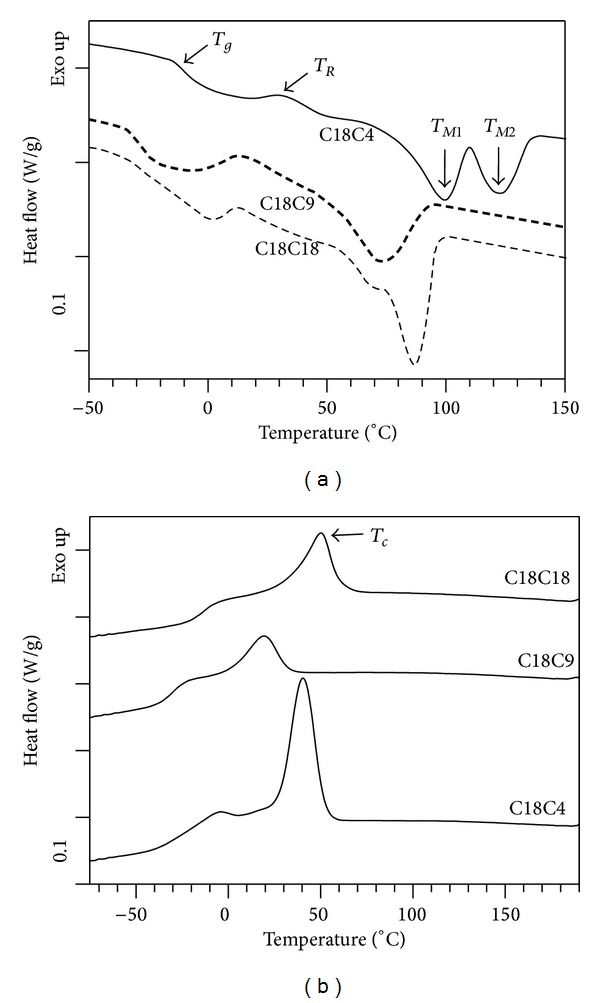
DSC heating (a) and cooling (b) thermograms of C18C18, C18C9, and C18C4 polymers. *T*_*g*_ = glass transition temperature, *T*_*R*_ = recrystallization temperature, *T*_*c*_ = crystallization temperature, and *T*_*M*1_ and *T*_*M*2  _= peak melt temperatures.

The DSC heating profiles (5°C/min) of the polymers, followed by the subsequent cooling (5°C/min), are shown in Figure 4(b) and the corresponding thermodynamic data are listed in Table 1. The heating thermograms of the polymers (Figure 4) revealed four transitions: a glass transition, then an exothermic recrystallization, followed by melting endotherms. C18C4 and C18C18 presented two well-resolved melting peaks and C18C9 exhibited convoluted peaks. One can note that C18C9 and C18C18 presented similar glass transition temperatures (*T*_*g*_ = −30.0 ± 1.6°C), recrystallization temperatures (*T*_*R*_ = 12.0 ± 1.7°C and 11.6 ± 0.4°C, resp.), and peak temperatures of melting (*T* = 65.8 ± 0.7°C and 64.4 ± 0.2°C, resp.). However, peak temperature of the second melt was relatively higher in C18C9 (*T*_*M*2_ = 86.5 ± 0.1°C), compared to C18C18 (*T*_*M*2_ = 73.5 ± 1.2°C). The enthalpy of the first and second endotherms in C18C9 was 6.0 ± 0.8 and 14.1 ± 0.2 J/g, respectively, and in C18C18 was 3.0 ± 0.3 J/g and 30.5 ± 0.9 J/g, respectively. The peak temperature of the two endotherms in C18C4 were at 97.5 ± 1.4 and 121.3 ± 1.5°C; significantly higher than in C18C9 and C18C18.

The presence of two melting peaks is often observed in polymers upon cold crystallization or crystallization from the melt [34]. In polyurethanes, the melt recrystallization forms an incomplete, poorly organized crystalline polymorph or liquid crystal [35]. The enthalpy measured for the first endotherm of all of the polymers was approximately equal to the enthalpy of the recrystallization peak suggesting that the lower melting peak (*T*_*M*1_) corresponds to the portion which has recrystallized. The enthalpy of the second endotherm was roughly equal to the enthalpy measured for the exotherm obtained during the crystallization from the melt (cooling rate of 5°C/min) in each polymer (Table 1). This indicates that the second endotherm is attributable to the melting of a more stable phase that has been formed during the cooling from the melt of the sample. Taking into account the odd-even effect, the melting point for the polymers of this study followed common trends observed in polyesters, where shorter monomer chain length imparts higher melting points [36]. The odd-even effect manifested in C18C9 where the number of methylene carbons between the two ester groups is odd resulting in the ester groups repeating symmetrically on the same side of the chain (Figure 2). This configuration limits the accessibility of hydrogen bonding, when compared to an even number of methylene atoms between the esters, placing “terminal groups” of each monomer on opposing sides. An explanation of this effect based on the odd-evens reduction in crystallinity has been previously used in polyesters, where monomers from odd-chain length exhibit reduced enthalpies of melt and crystallization [37]. The density of triazole hydrogen bonding within a polymer has previously been demonstrated to be a factor influencing the glass transition temperature of polymers and dictating many thermal properties as well [38]. In the study by Binauld et al., monomers with equal carbon numbers were synthesized with both ester and ether linkages then polymerized into polytriazoles containing only ether linkages, polytriazoles with ester linkages only, and a copolymer consisting of both. They reported that thermal properties including melt temperature and glass transition were minimally affected by exchanging the ester and ether groups, suggesting that the triazole group was predominantly dictating the polymers' thermal properties [38]. Our findings suggest that an odd length chain in a polytriazole significantly reduces the melt and crystallization enthalpies of that polymer when compared with even length chains.

### 4.3. Crystal Structure of the Polymers

**Figure 5:**
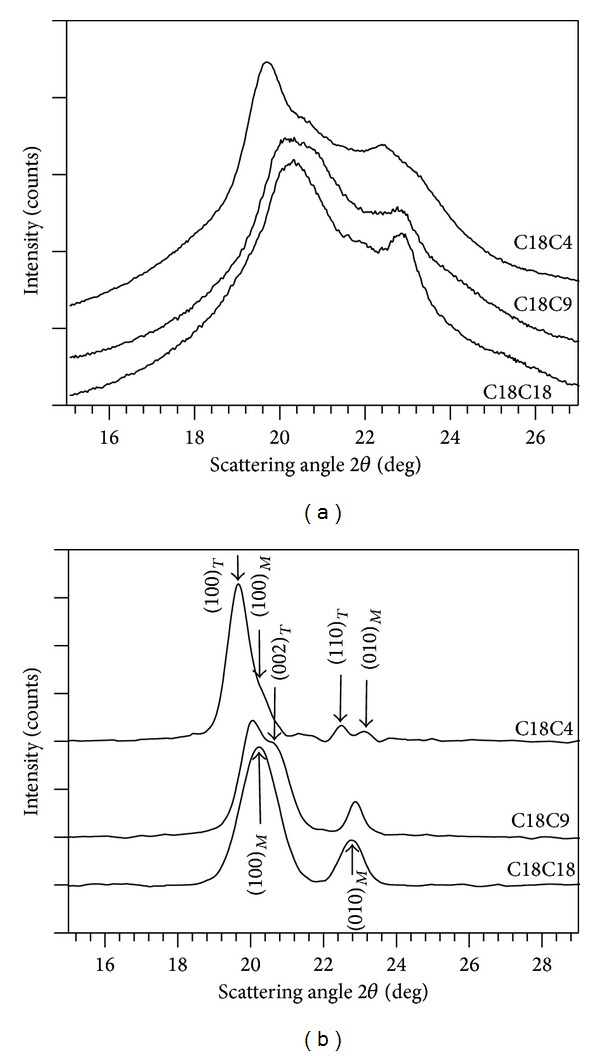
(a) Representative WAXD spectra of the polymers at room temperature and (b) crystal contributions to the WAXD spectra after subtracting the baseline and amorphous contributions. Subscripts *T* and *M* of the Miller indices (hkl) are for the triclinic and monoclinic structures, respectively.

WAXD spectra of the polymers are shown in Figure 5(a) and corresponding d-spacing and Miller indices of the crystal reflections are listed in Table 2. As can be seen, the experimental WAXD profiles of all the polymer samples consisted of resolved diffraction peaks superimposed to a relatively large wide halo, indicative of the presence of an amorphous phase. This type of WAXD patterns is commonly observed in semicrystalline polyurethanes [39]. The amorphous contribution was subtracted using HighScore V3.0.4 software and the resulting WAXD spectra of the polymers with only the crystal peaks are shown in Figure 5(b). The WAXD lines were indexed using data obtained from similar compounds found in the literature [2, 40].

C18C18 presented two lines at 4.40 Å (100) and 3.91 Å (010), characteristic of the monoclinic subcell, similarly to what was reported for vegetable oil derived poly (ester-urethanes) [2]. C18C9 presented the two monoclinic peaks detected in C18C18 at 4.44 Å (100), 3.89 Å (010), and an additional peak at 4.30 Å (002), a reflection associated with a triclinic subcell [40]. In C18C4, the two monoclinic peaks slightly shifted to 4.37 Å (100) and 3.86 Å (010) and two new reflections from the triclinic phase appeared at 4.52 Å (100) and at 3.96 Å (110) [2, 40]. The data obtained for the crystalline phases of the polymers are consistent with the proposed bonding structures. C18C18 shows a single crystal structural phase compatible with predominantly hydrogen bonding, whereas, C18C9 and C18C4 showed coexisting triclinic and monoclinic crystalline phases which can be related to the two bonding types suggested for these two polymers.

### 4.4. Thermal Gravimetric Analysis

**Figure 6:**
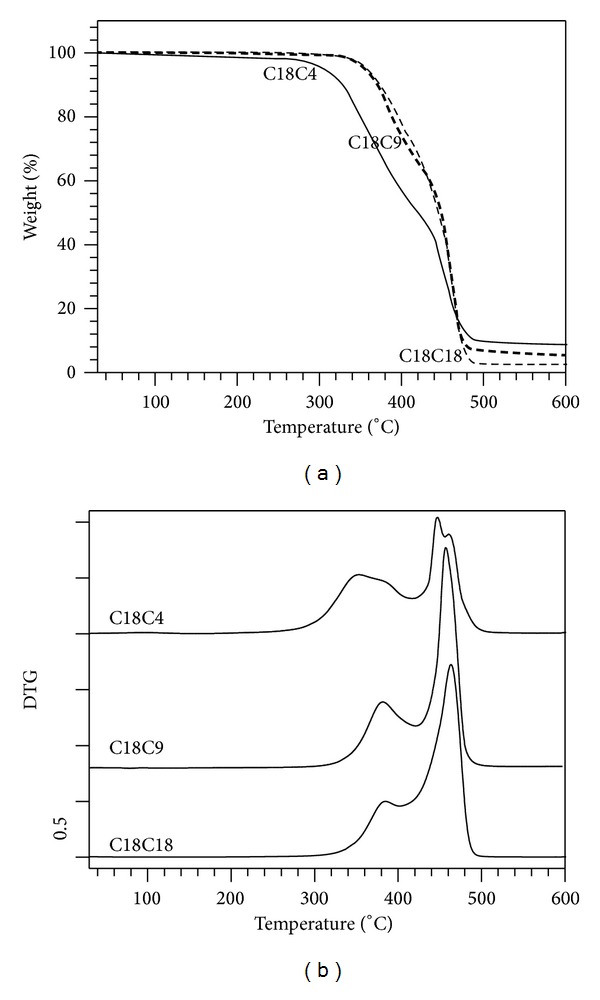
(a) TGA and (b) DTG curves of C18C18, C18C9, and C18C4 polymers.

TGA and DTG curves of the polymers are shown in Figures 6(a) and 6(b), respectively. C18C18 and C18C9 exhibited very similar degradation profiles characterized by a DTG with two well-resolved peaks indicative of a two-step degradation process. Both polymers presented onsets of degradation (at 5% weight loss) at ~360°C and maximum rate of weight loss (DTG peak) at approximately 460°C. The onset of degradation of (5% weight loss) of C18C4 was measured at 306°C, a much lower temperature than the two others. Furthermore, its DTG presented four resolved peaks, two of which corresponded to those observed in C18C9 and C18C18 and two others at 352°C and 446°C. The DTG of C18C4 in fact appeared to be the superimposition of two similar thermal degradation profiles, one occurring after the other. This suggests the presence of two phases which, although degrading through the same mechanisms, did involve different atomic environments.

The first decomposition peak in the DTG (Figure 6(b)) for C18C4 is broad and represents a higher area than the other two, due to the shorter chain length and greater ester contribution to the polymers' weight. This peak is commonly associated with ester degradation in polyesters [2]. When the two ester groups are closest to each other, as in C18C4, the electron withdrawing effect of the ester groups in close proximity to the electronegative triazole is strongest on the 2 *α*-carbon atoms between the esters [41]. This may be responsible for the more rapid degradation.

### 4.5. Mechanical Properties

**Figure 7:**
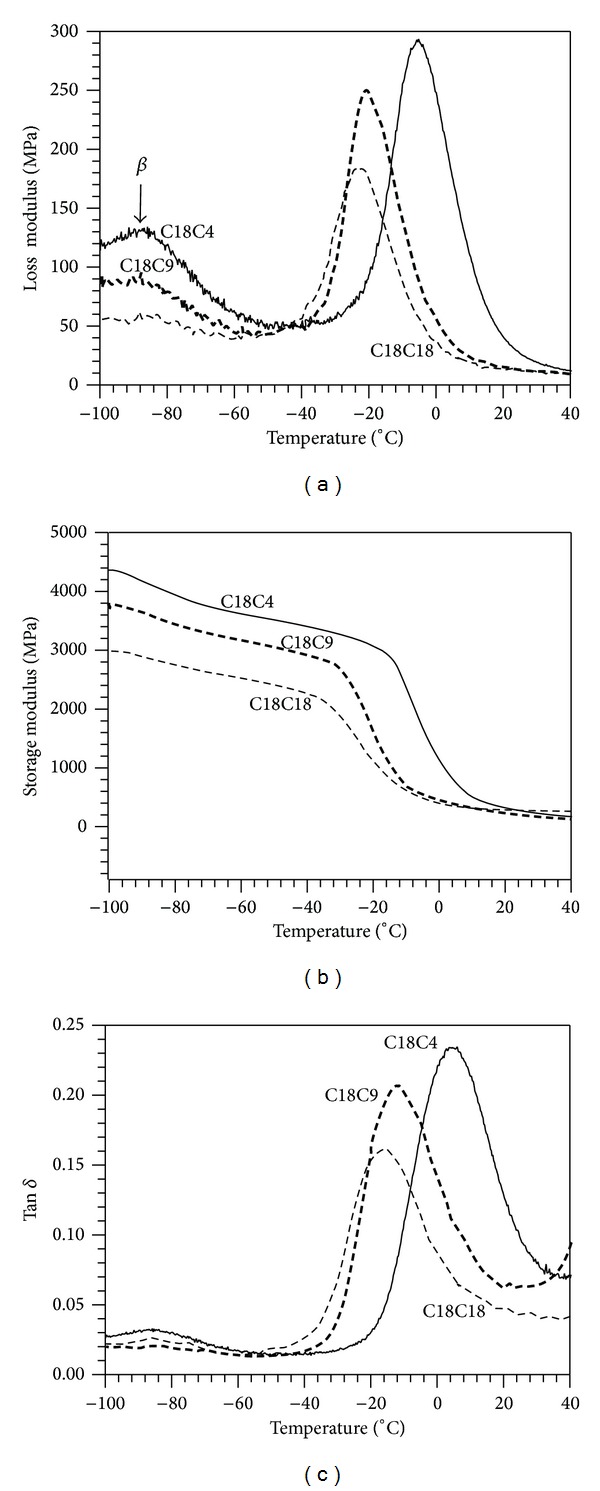
Representative DMA results of C18C18, C18C4, and C18C9 polymers. (a) Loss modulus; (b) storage modulus; and (c) tan *δ*.

The viscoelastic properties of the polytriazoles were studied using DMA. The loss modulus, storage modulus, and tan *δ* of the polymers are shown in Figures 7(a) to 7(c), respectively. Loss modulus and tan *δ* versus temperature curves indicated homogeneous systems with single glass transitions. The prolonged glass transition observed by DSC is probably due to enthalpy of relaxation effects. The first peak observed at ~−85°C is attributable to the so-called *β* transition. As clearly seen in the loss modulus graph in Figure 7(a), the *β*-transitions in C18C4 were much more intense than C18C9 and C18C18 where it was very weak. This transition may be attributed to the limited mobility of the ester carbonyl group, or the shorter methylene chain segments, both of which increase through the series from *n* = 4 to 18 [42, 43]. This would explain the reduction and disappearance of the peak with increasing *n*; as the chain length increased, its mobility would decrease.

In Figure 7(b), the storage modulus in the glassy and leathery regions are the highest value for C18C4 and lowest for C18C18. In the rubbery region, storage modulus is the same for the three polymers. The sharp drop in the storage modulus around −40 to −10°C represents the beginning of the transition from the glassy to rubbery state. The *T*_*g*_ of C18C18 and C18C9, as determined from the peak tan *δ* curve, Figure 7(c), was found to be very close (−16.4 ± 0.2 and −12.8 ± 0.8°C) and significantly lower than that of C18C4 (6.6 ± 1.1°C, Table 3). The trends observed for *T*_*g*_ as measured by DMA can be explained in terms of the structural differences between the polymers and related to the competing effects of the hydrogen bonding introduced by the triazole groups and the esters contributions which in fact determine a “hydrogen bond density and distribution”. Note that the trends in the *T*_*g*_ obtained by DMA in Table 3 mirrored those recorded by DSC, although the DMA was more resolved as it was able to detect a *T*_*g*_ difference between C9 and C18.

**Figure 8:**
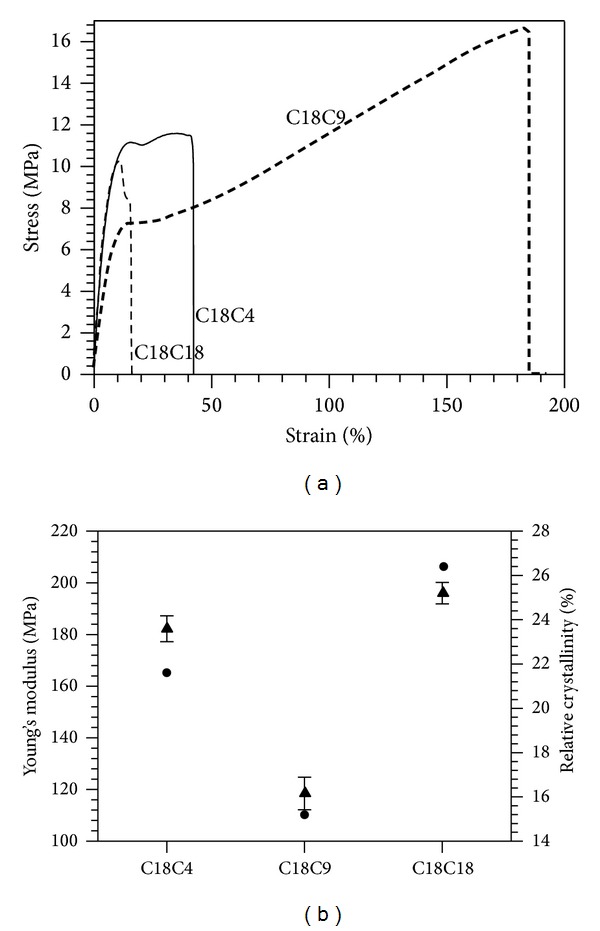
(a) Representative stress strain curves for of C18C18, C18C4, and C18C9 polymers. (b) Young's modulus (●) and relative crystallinity (▲) of the polymers. Error bars represent standard deviation from at least triplicates.

Stress-strain curves of the polymers are shown in Figure 8(a). Tensile properties range from relatively brittle to ductile thermoplastics, with elongation to break percentages comparable to LDPE for C18C9. Young's modulus (E) closely followed the degree of crystallinity of the polymer. In fact, as shown in Figure 8(b) representing both *E* and *X*_*C*_, the two parameters are strongly correlated. This is not surprising as the effect of crystallinity on polymers Young's moduli is well known [44]. All three of the polymers in this study have Young's moduli comparable with LDPE (Table 4).

The elongation at break of the less crystalline C18C9 was 196.6 ± 16.5%: much higher than C18C4 at 39.6 ± 3.8% and C18C18 at 10.7 ± 3.8% (Table 4). This result can be explained by differences in hydrogen bonding and crystallinity. A lower hydrogen bonding density caused by the odd-even effect results in decreased crystallinity in C18C9 (Figure 8(b)).

### 4.6. The Odd-Even Effect on the Physical Properties

As the predominating hydrogen bonding mechanism shifts from triazole self-association to triazole—carbonyl on shortening of the diester chain segment, a transition in properties is observed, with C18C9 presenting mid-way characteristics. FTIR data suggests that the hydrogen bonding from triazole-triazole and triazole-ester hydrogen bonding is reduced in C18C9 compared to C18C18 and C18C4. The crystallinity of the C18C9 polymer was also significantly reduced. Previous studies have shown that odd-carbon length monomers can prevent up to 50% of the hydrogen bonding interactions due to altered orientations [45]. An odd carbon number can also create repulsive interactions between the flipped carbonyl groups with the chain due to the close stacking of the ester groups [45]. In contrast, an even-carbon length monomer allows the groups on each terminus to be in opposite relative orientations, allowing more ordered hydrogen bonding networks and more favourable packing [45]. The odd-even effect's influence in crystallinity has also been well documented in polyurethanes [46]. Similar findings for a variety of hydrogen bonding systems, including nylon [47], glucoseamide polymers [48], polycarbonate [45], fatty acids [49], fatty alcohols [50], and polyesters [37], describe similar trends. The findings of this study are consistent with the polymer chains of C18C9 being adapted into contracted, sterically unfavourable configurations forming disordered or reduced hydrogen bonding, similar to what has been reported for nylon [45] and polyurethane [46]. The diester dialkynes with odd carbon number chain length form polymers with both the ester and triazole groups on the same side, altering the degree of crystallinity, melting and crystallization temperatures, glass transition temperatures and mechanical properties.

## 5. Conclusions

Three novel thermoplastic polytriazoles were produced by uncatalyzed and solvent free polymerization of vegetable oil derived diester alkyne and diazide monomers. The three dialkynes were synthesized from economically priced succinic acid (C4), azelaic acid (C9), and oleic acid (C18). The diazide monomer, prepared from C18 diacid, had a ratio of 3 carbon to 1 nitrogen atoms; generally accepted as sufficient to prevent the rapid decomposition or explosion of azide groups and to allow safe storage and processing.

The monomers and polymers were fully characterized by NMR and ATR-FTIR. Crystal structure, thermal behavior, and mechanical properties of the polymers, measured using XRD, TGA, DSC, DMA, and tensile test, were related to the structural details of the repeating unit, compared and contrasted to the paper. The trends observed in the thermal behaviour and mechanical properties of the polymers were explained based on differences in their crystallinity and ability to hydrogen bond. The polymers synthesized from even chain length monomers had significantly higher crystallinity, higher melting points and melt enthalpy, higher Young's modulus values, and lower elongation-to-break percentages when compared with the odd chain length monomer. The crystallinity and melt and crystallization enthalpies of C18C9 were approximately half of those of the other polymers. The odd-even effect, which dictates ordering of groups in symmetrical or asymmetrical configurations based on the monomer chain length, was used to explain this behaviour. The differences observed in physical property between the polymers are consistent with the presence of two different hydrogen bonding systems: hydrogen bonding between ester—triazole groups, and triazole—triazole groups. C18C18 was suggested to have predominantly ester—triazole hydrogen bonding, C18C4 predominantly triazole—triazole hydrogen bonding and the third polymer, C18C9, which has an odd carbon length monomer, a mixture of these two hydrogen bonding systems. This work advances effective routes for “green” polymerization and provides a catalyst and solvent free method to make functional thermoplastic polymers from vegetable oils. This opens the possibility of future use in applications sensitive to catalyst or solvent contamination, including medical and pharmaceutical applications.

## Supplementary Material

A magnified proton NMR spectra of the polymer C18C9 from Figure 1, showing enhanced resolution of the triazole region shifts is provided in Figure S1 in the Supplementary Material.

## Figures and Tables

**Table 1:** Thermodynamic data obtained from the DSC cooling and heating cycles of C18C18, C18C9, and C18C4 polymers.

Polymer	*T*_*C*_ (°C)	Δ*H*_*C*_ (°C)	*T*_*m*1_ (°C)	*T*_*m*2_ (°C)	Δ*H*_*m*1_ (J/g)	Δ*H*_*m*2_ (J/g)	*T*_*R*_ (°C)	Δ*H*_*R*_ (J/g)
C18C4	50.9 ± 0.4	27.6 ± 4.4	97.5 ± 1.4	121.3 ± 1.5	12.9 ± 1.5	14.1 ± 0.2	12.0 ± 1.7	6.1 ± 1.0
C18C9	20.0 ± 1.1	13.9 ± 0.2	65.8 ± 0.7	73.5 ± 1.2	6.0 ± 0.8	—	—	—
C18C18	40.8 ± 0.8	32.4 ± 2.8	64.4 ± 0.2	86.5 ± 0.1	3.0 ± 0.3	30.5 ± 0.9	11.6 ± 0.4	3.5 ± 0.5

Crystallization temperature (*T*_*C*_) and crystallization enthalpy (Δ*H*_*C*_); melting temperatures (*T*_*m*1_ and *T*_*m*2_) and enthalpy of melt (Δ*H*_*m*1_ and Δ*H*_*m*1_), temperature of recrystallization *T*_*R*_, and enthalpy of recrystallization (Δ*H*_*R*_). Values and uncertainties attached are, respectively, average and standard deviation of triplicates.

**Table 2:** WAXD data obtained for C18C18, C18C9, and C18C4 polymers at room temperature.

Polymer	Monoclinic phase		Triclinic phase		Relative crystallinity (%)
	hkl	*d* (Å)	hkl	*d* (Å)	
C18C4	(100)	4.37	(100)	4.52	20.6
	(010)	3.86	(110)	3.96	

C18C9	(100)	4.44	(002)	4.30	15.1
	(010)	3.89			

C18C18	(100)	4.40	—	—	26.3
	(010)	3.91			

*d*: Bragg distances; (hkl): corresponding indices.

**Table 3:** Glass transition temperature (*T*_*g*_), as determined by DSC and DMA.

Polymer	*T*_*g* (DSC)_ (°C)	*T*_*g* (DMA)_ (°C)
C18C4	−11.8 ± 0.7	6.6 ± 1.1
C18C9	−30.0 ± 1.3	−12.8 ± 0.8
C18C18	−30.0 ± 1.6	−16.4 ± 0.2

Values and uncertainties attached are respective average and standard deviations of triplicates.

**Table 4:** Mechanical properties of the polymers in this study compared to three common commercial polymers.

	Young's modulus (MPa)	Elongation at break (%)	Ultimate tensile strength (MPa)
Polymers in this study			
C18C4	182.2 ± 5.0	39.6 ± 3.8	11.1 ± 0.4
C18C9	118.5 ± 6.3	196.6 ± 16.5	14.8 ± 1.5
C18C18	196.0 ± 4.1	10.7 ± 3.8	10.3 ± 0.5
Polymers for comparison			
Commercial LDPE [25]	140–300	200–900	7–17
Vegetable Oil derived Cross-linked polytriazole [6]	Not Reported	31.1–61.0	0.62–3.39
Vegetable Oil derived thermoplastic polyurethane [26]	0.7–11.1	25–600	1.3–5.6

Values and uncertainties attached are, respectively, average and standard deviation of triplicates.
