# Supplementary material for: Vegetable Oil Derived Solvent, and Catalyst Free “Click Chemistry” Thermoplastic Polytriazoles
Source: Biomed Res Int. 2014 Jun 17;2014:792901. doi: 10.1155/2014/792901 (PMC4085725; doi:10.1155/2014/792901)
Supplement: Supplementary file 1 — A magnified proton NMR spectra of the polymer C18C9 from Figure 1, showing enhanced resolution of the triazole region shifts is provided in Figure S1 in the Supplementary Material. [file 792901.f1.pdf]

Supplementary Information

Vegetable Oil-Derived, Solvent and Catalyst Free 'Click Chemistry' Thermoplastic Polytriazoles

Michael C. Floros [†], *Alcides Lopes Leão* [‡], *and Suresh S. Narine* [†]*

[†] Trent Centre for Biomaterials Research, Departments of Physics & Astronomy and Chemistry, Trent University, Peterborough, Canada.

[‡] College of Agricultural Sciences, São Paulo State University, (UNESP), Botucatu, Brazil.

*Trent University, Peterborough, Canada. Fax: 70 5750 2786; Tel: 70 5748 1011; E-mail: sureshnarine@trentu.ca

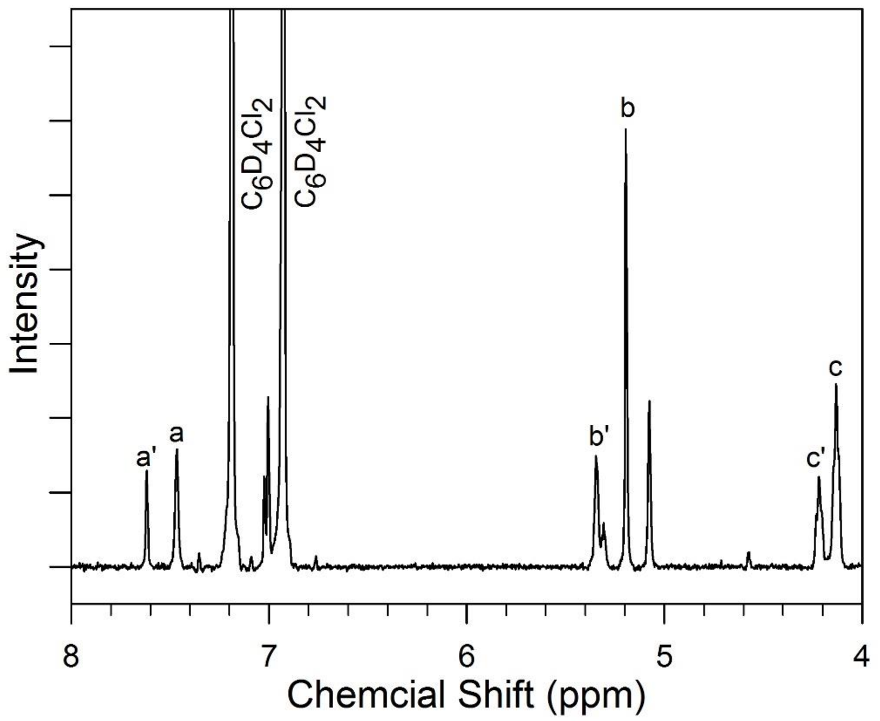

Figure S1. Zoom on Figure 1.
